# Fabrication and Characterization of Buforin I-Loaded Electrospun Chitosan/Polyethylene Oxide Nanofibrous Membranes with Antimicrobial Activity for Food Packing Applications

**DOI:** 10.3390/polym17040549

**Published:** 2025-02-19

**Authors:** Sahar Roshanak, Hanieh Yarabbi, Jebraeil Movaffagh, Fakhri Shahidi

**Affiliations:** 1 Department of Food Science and Technology, Faculty of Agriculture, Ferdowsi University of Mashhad, Mashhad 9177948974, Iran; s.roshanak@um.ac.ir (S.R.); hyarabbi@um.ac.ir (H.Y.); 2 Department of Pharmaceutics, School of Pharmacy, Mashhad University of Medical Sciences, Mashhad 1394491388, Iran

**Keywords:** chitosan, Buforin I, electrospinning, antimicrobial peptide, antimicrobial membrane, shelf life extending

## Abstract

The rising resistance of bacteria to antibiotics has driven the search for new antimicrobial agents. This study focused on encapsulating Buforin I, an antimicrobial peptide, in chitosan/polyethylene oxide (CS-PEO) nanofibers. Buforin I was loaded at a minimum bactericidal concentration (MBC), 10× MBC, and 20× MBC, with assessments on morphology, thermal properties, chemical bonds, crystalline structure, mechanical strength, antimicrobial activity, and cell toxicity. Techniques like differential scanning calorimetry and Fourier-transform infrared spectroscopy confirmed the effective loading of Buforin I in the nanofibers. Scanning electron microscopy showed that Buforin incorporation increased nanofiber diameters. The tensile strength peaked at 20× MBC. Microbial tests indicated that the inhibition zone for nanofibers at 20× MBC surpassed that of commercial antibiotics. Beef coated with CS-PEO nanofibers containing Buforin I demonstrated reduced pH and water activity, alongside lower weight loss during storage. Texture and color analyses revealed that the Buforin I nanofibers helped maintain beef hardness and slowed color degradation compared to control samples. Moreover, thiobarbituric acid levels and total microbial counts in the coated beef were significantly lower than controls (below 3 log CFU/g after 9 days at 4 °C). Thus, these nanofibers may serve as effective antimicrobial packaging agents to delay food spoilage.

## 1. Introduction

As a global issue, food safety has important implications for human health. Rising concerns about synthetic preservatives, the prevalence of antibiotic-resistant food pathogens, and the increasing consumer demand for natural and minimally processed foods have caused many technological challenges in the food industry [1]. The use of recombinant proteins in preventing the growth of pathogens has received special attention in food safety, and effective steps have been taken in this area. Biologically active peptides are important in regulating and modulating the cellular metabolic system [2]. They can be used as functional components, nutraceuticals, and drugs to inhibit disease and improve human health. Based on their structural properties and amino acid composition and sequence, such peptides may be active in various biological functions such as inhibiting the activity of the angiotensin-I-converting enzyme (ACE), strengthening the immune system, as well as antimicrobial, anticoagulant, and antioxidant activities [3]. Cationic antimicrobial peptides (AMPs) are produced against infections by almost all organisms as a part of their non-specific defense barrier [4].

Histone-derived peptides are a class of antimicrobial peptides that do not interfere with replication. Histone extracellular derivatives have strong antimicrobial properties [5]. As a histone extracellular derivative, Buforin I is a peptide containing 39 amino acids, isolated from the stomach tissue of *Bufo bufo gargarizans*, an Asian toad [6]. Buforin I is capable of inhibiting the growth of a variety of Gram-positive bacteria (*Bacillus subtilis*, *Staphylococcus aureus*, *Streptococcus mutans*, *Streptococcus pneumoniae*, and *Pseudomonas putida*), Gram-negative bacteria (*Escherichia coli*, *Salmonella typhimurium*, and *Serratia* species), and molds (*Candida albicans*, *Cryptococcus neoformans*, and *Saccharomyces cerevisiae*) [7].

The low stability of natural bioactive compounds in environmental conditions causes their total or partial degradation, which reduces their efficiency and limits their applications. The antimicrobial activity of Buforin I may be impaired, due to its susceptibility to proteolytic degradation and adverse interactions. Encapsulation is an efficient method for increasing bioactive compounds’ stability. The microencapsulation of antimicrobial peptides raises their resistance to high temperature and acidic conditions and improves their antimicrobial activity against Gram-negative and Gram-positive bacteria [8]. Electrospinning is one of the ideal methods for the encapsulation of environmentally sensitive compounds, due to the use of ambient temperature throughout the process. Electrospun nanofibers are considered for various biomedical applications or the production of antimicrobial membranes. They can be an interesting option for delaying food surface spoilage [9]. Nanofibers have a large specific surface area in addition to high porosity, loading capacity, and encapsulation efficiency [10]. This study was conducted according to previous research on the Buforin I peptide. Previous results have shown that this peptide had high antimicrobial effects [7].

Meat contains high-quality protein and essential micronutrients. It is very sensitive to lipid oxidation reactions and microbial contamination of *E. coli*, *Salmonella*, *Staphylococcus aureus*, and *Listeria monocytogenes*. The off odors in oxidized meat are mainly caused by aldehydes, especially hexanal [11]. Edible coatings play an important role in preventing the movement of solutes, moisture, and oxygen in foods, which have been noted for their excellent performance in food preservation, especially for meat packaging. Edible coatings containing antimicrobial food ingredients effectively control the growth of microorganisms, increasing the shelf life of meat products [12]. Therefore, this study aimed to analyze the electrospun encapsulation of Buforin I using chitosan (CS) and polyethylene oxide (PEO) and evaluate the characteristics of the produced nanofibers. Following this, the effectiveness of a Buforin I-based edible coating to increase the shelf life of fresh beef during storage at 4 °C was investigated.

## 2. Materials and Methods

Polyethylene oxide (molecular weight 600,000 to 1,000,000 g/mol) and gallic acid were purchased from Sigma Aldrich, St. Louis, MO, USA. Low molecular weight chitosan (molecular weight 161.16 g/mol, Cat No: ABC11-P250) was supplied by Atomix (Trahutten, Austria). Buforin I was synthesized by the TAG Copenhagen Company (Frederiksberg, Denmark) using the amino acid sequence available from the NCBI database (https://www.ncbi.nlm.nih.gov/, accessed on 19 June 2024), with accession number: P55897. Fresh beef slices were purchased from a local market in Mashhad, Iran. All chemicals were used without further purification and all chemical reagents were of analytical grade.

### 2.1. Preparation of Electrospinning Solution

The electrospinning solutions were prepared based on the optimized conditions in our laboratory, reported by Kalalinia et al. (2021) [13]. According to our previous studies, different concentrations of Buforin I were adjusted based on a higher MBC observed for more than 20 microbial species [13,14,15]. A total of 1, 10, and 20 times the minimum bactericidal concentration (MBC = 16 µg/mL) were added to the solution composed of CS (2.5%, *w*/*w*) and PEO (2.5%, *w*/*w*). This solution was placed overnight on a magnetic stirrer at 40 rpm. Then, it was debubbled by sonication [13].

### 2.2. Measurement of Viscosity and Electrical Conductivity of Electrospinning Solutions

The viscosity of the solutions was measured by a rheometer (Brookfield RV DVIII Ultra, East Lyme, CT, USA). Electrical conductivity was also determined using a portable digital conductivity meter (8302 AZ, Goldpoint Co., Taichung, Taiwan).

### 2.3. Electrospinning

Nanofibers were prepared using a horizontal two-axis electrospinning device (NanofanAvaran Co., Tehran, Iran) at 0–35 kV. The nozzle was made of stainless steel with an inner diameter of 0.9 mm (18 G). The operation factors were kept constant as follows: nozzle-collector distance of 150 mm, drum rotation speed of 150 rpm, pump feed rate of 0.5 mL/h, and applied voltage of 12.5 kV [16,17].

### 2.4. Scanning Electron Microscope (SEM) Imaging

The morphology of the samples after atomic bombardment with a mixture of gold and palladium in a vacuum was assessed using SEM (LAO-1450VP, Freising, Germany), and their average diameter was determined by Image J (1.54b 8 January 2023).

### 2.5. Differential Scanning Calorimetry (DSC) Analysis

Differential scanning calorimetry analysis for buforin I in a free state and electrospun fibers was performed using a DSC device (Dazhan, Nanjing, China). Enthalpy (area under the peak), starting point, and maximum peak, as well as Tg, were calculated.

### 2.6. Fourier Transform Infrared (FTIR) Spectroscopy

Functional groups were identified and the type of bonds established between chitosan, Buforin I, and polyethylene oxide in electrospun fibers were determined using a Thermo Nicolet spectrophotometer model AVATAR 370 FTIR at 350–400 cm^−1^.

### 2.7. X-Ray Diffraction

XRD analysis was performed for Buforin I and for fibers obtained from electrospinning. The test was performed at 40 kV and 30 mA using XRD model Explorer, Italy.

### 2.8. Mechanical Resistance Test of Nanofibrous Membranes

Electrospun fibers with dimensions of 1 × 4 cm^2^ and a specified thickness were placed between two clamps of a Hounsfield H50SK machine (Redhill, UK). Then, the elasticity test was performed at the specified speed. After recording the graph, the Young’s modulus and yield point were determined.

### 2.9. Loading Efficiency of Peptide in Nanofibrous Membranes

A 2 × 2 cm^2^ piece was prepared from each of the electrospun fibers. The amount of peptide was calculated theoretically. Then, these parts were placed in a 1.5 mL microtube and 1 mL of 80% acetic acid was added to them until the membrane was dissolved. The peptide concentration of each sample was calculated using a standard curve. The peptide loading percentage was determined using the following equation [17]:

Loading percentage = (actual value)/(theoretical value) × 100

### 2.10. Cumulative Release of Peptides from Nanofibrous Membranes

A 2 × 2 cm^2^ piece of the parts was placed in a 6-well microplate along with 1 mL of PBS buffer with pH = 7.4. They were incubated in a shaker incubator at 37 °C and 80 rpm. At 10 h intervals for 150 h, the absorption of the samples was evaluated using UV-visible spectrophotometry UVD-2950, LABOMED (Los Angeles, CA, USA) at 205 nm and compared with the standard curve [17].

### 2.11. Release Kinetics of Buforin I from Nanofibrous Membranes

The release kinetics for Buforin I-loaded nanofibers were obtained by the Korsmeyer–Peppas equation.

Mt/M∞ = Ktn

where Mt is the amount of Buforin I released at time t, M∞ is the total amount of Buforin I in the nanofibers, K is the kinetic constant, and n is the release exponent, which could be used to determine the release mechanisms [17].

### 2.12. Water Absorption Capacity of Nanofibrous Membrane

The weight of a cut of 2 × 2 cm^2^ from each of the electrospun fibers was determined (W_0_). After 24 h, the weight of each of them was measured (W). The following formula is used to calculate the percentage of water absorbed [18]:

WC (%) = ((W − W_0_)/W_0_) × 100

### 2.13. Water Vapor Transfer Capacity of Nanofibrous Membrane

The samples (2 × 2 cm^2^) were fixed on a 15 mL falcon with an opening diameter of 17 mL containing 5 mL of distilled water. The falcon was incubated in a 37 °C incubator for 24 h. The WVTR was calculated based on the following formula, where ∆W is the weight change of the falcon and A is the open surface area of the falcon [18]:

WVTR = ∆W/A

### 2.14. Antimicrobial Activity of Nanofibrous Membrane

The antimicrobial activity of the peptide-loaded nanofibers was assessed through agar disk diffusion in MHA against *S. aureus* (ATCC25923), *E. coli* O157:H7 (ATCC 35150), *L. monocytogenes* (PTCC1297), *S. Typhi* (PTCC 1609), *B. cereus* (PTCC1247), *P. aeruginosa* (PTCC 1707), *C. albicans* (PTCC 5027), and *A. niger* (PTCC 5010). Therefore, three disks with a diameter of 8 mm were prepared from each of the nanofibers, and both sides of the disks were sterilized by being exposed to UV light for 30 min [19]. Afterward, 100 µL of the microbial suspensions were spread onto the surface of MHA or PDB, and the sterilized nanofiber punched was placed on it, followed by incubation at 37 °C for 24 h or 25 °C for 75 h, for bacterial or fungal strains, respectively. The inhibition zone diameter surrounding the disk was measured with a ruler in millimeters. The peptide-free nanofibers and commercial antibiotic disks were used as negative and positive controls, respectively. The commercial antibiotic disks for Gram-positive bacteria, Gram-negative bacteria, and molds were comprised of vancomycin (30 µg), tetracycline (30 µg), and nistatin (50 µg), respectively [15].

### 2.15. Hemolytic Activity of Nanofibrous Membrane

A total of 4 mL of freshly prepared human red blood cells (RBCs) were mixed with 50 µL of EDTA and centrifuged (2422 g, 10 min). The residue was dissolved in 4 mL of PBS buffer and centrifuged (2422 g, 10 min) successively until the color of the supernatant turned clear. The washed RBCs were then diluted to a final volume of 45 mL with the same buffer. A total of 200 μL of the prepared blood suspension was poured into seven 1.5 mL microtubes, and a sterile nanofiber disc was added to five microtubes. The two remaining microtubes were treated with 20 µL of PBS buffer (as negative control) and Triton X100 (as positive control), respectively. Following gentle mixing, the tubes were incubated at 37 °C for 30 min and then centrifuged (1914 g, 30 min). A total of 100 µL of the supernatant was taken, diluted to 1 mL with PBS buffer, and its absorbance value was measured at 570 nm to monitor the release of hemoglobin that indicates the RBC membrane damage [14]. The percentage of hemolysis was determined as follows:

Hemolysis (%) = (A_S_ − A_0_)/(A_100_ − A_0_) × 100

where A_s_ is the absorbance of the sample, A_0_ is the absorbance of the negative control sample, and A_100_ is the absorbance of the positive control sample. The highest peptide concentration that does not cause hemolysis was defined as the minimum hemolytic concentration (MHC) [14].

### 2.16. Cytotoxic Assay of Nanofibrous Membrane

Cytotoxicity was investigated using human dermal fibroblasts (HDFs) in Dulbecco’s Modified Eagle’s medium (DMEM), 100 unit/mL penicillin, 100 μg/mL streptomycin, and 10% fetal bovine serum (FBS). Circular disks (diameter, 6 mm) of the electrospun membrane with concentrations of 0, 50, 100, 200, 400, 600, 800, and 1000 μg/mL of peptide were placed in a 96-well culture plate and sterilized with plasma. Then, 5 × 10 cells/well were seeded on electrospun samples and the plate was incubated at 37 °C, 95% humidity, and 10% CO_2_ for 72 h. The cells were seeded in control samples with only DMEM medium with 10% FBS, 100 units/mL penicillin, and 100 μg/mL streptomycin. Cell viability was evaluated by the resazurin reduction method and absorbance at 640 nm. Cell viability was calculated using the following formula:

Cell viability% = [(A_S_ − A_0_)/(A_C_ − A_0_)] ×100

where A_S_ is the absorption of the sample, A_C_ is the absorption of the control sample, and A_0_ is the absorption of the blank [14].

### 2.17. Beef Coating

Fresh beef was cut into pieces of 6 × 6 × 2 cm^3^. The treatments were prepared as uncoated beef (control sample), beef coated with CS-PEO nanofibers without Buforin I, and meat coated with CS-PEO nanofibers with Buforin I. Then, treatments were put in zip bags and moved into the refrigerator (5 °C). The samples were examined on days 0 (on the same day of coating), and 3, 6, and 9 days after storing in the refrigerator [19].

### 2.18. pH, Weight Loss, and Water Activity (a_w_) Determination

The beef slices were homogenized using a blender, and their pH values were measured by a pH meter (InoLab 7310, Weilheim, Germany) [20]. Coated beef pieces were weighed on days 0, 3, 6, and 9 to measure weight loss. The weight loss percentage was expressed as the lost mass of beef compared to its initial mass [21]. Water activity values of the samples were measured using water activity meter 3012-66 (Novasina, Tokyo, Japan) during the storage [22].

### 2.19. Texture Profile Analysis (TPA)

The texture properties of beef samples, i.e., hardness, springiness, cohesiveness, gumminess, and chewiness were measured by a Texture analyzer, model AMETEK Lloyd TA-Plus Instruments Ltd. (Eden Prairie, MN, USA). The samples of 20 × 20 × 20 mm were compressed up to 5% of the initial height. The penetration rate was 1 mm per second. The probe used was 100 N [23].

### 2.20. Thiobarbituric Acid (TBA)

The beef slices were evaluated for lipid oxidation by measuring thiobarbituric acid reactive substances (TBARSs) based on the method proposed by Dini et al. (2020) [24]. The sample (5 g) was mixed with a trichloroacetic acid (TCA) solution (7.5% *v*/*v*) (25 mL) containing Ethylenediaminetetraacetic acid (EDTA) (0.1% *m*/*v*) and homogenized using a blender. Then, the samples were centrifuged for 10 min at 5000 rpm. The supernatant was mixed with TBARS reagent (1% thiobarbituric acid, 562.5 μM, HCl, 15% TCA) (1:1 *v*/*v*). The mixture was boiled for 15 min, cooled, and analyzed for optical absorbance at 535 nm using a standard curve. The TBARS values were reported as mg malondialdehyde/kg of meat. Lipid oxidation was measured on days 0, 3, 6, and 9 [24].

### 2.21. Color Analysis

The color values (L*, a*, and b*) of the beef sample surfaces were detected using a HunterLab XE (Hunter AsSDSCiates Laboratory Inc. (Reston, VA, USA). Color was expressed as brightness (L*), redness (a*), and yellowness (b*). The analysis was performed in triplicate for each sample [25].

### 2.22. Microbiological Analysis

The microbial count of the beef samples was counted by the pour plate technique on days 0, 3, 6, and 9. Plate Count Agar, Eosin-Methylene Blue agar, Mannitol Salt Agar, and Salmonella-Shigella Agar were used for counting total aerobic bacteria, *E. coli*, pathogenic staphylococci, and pathogenic enteric bacilli, respectively. The microbial count results were reported as log CFU/g for meat samples [25,26].

### 2.23. Statistical Analysis

Minitab software 18.0 was used for the statistical analysis. A one-way ANOVA and *t*-test were used to compare between different groups. The Tukey–Kramer test was used to compare the average data.

## 3. Results and Discussion

### 3.1. Viscosity, Electrical Conductivity, and pH of Electrospinning Solution

**Figure 1:**
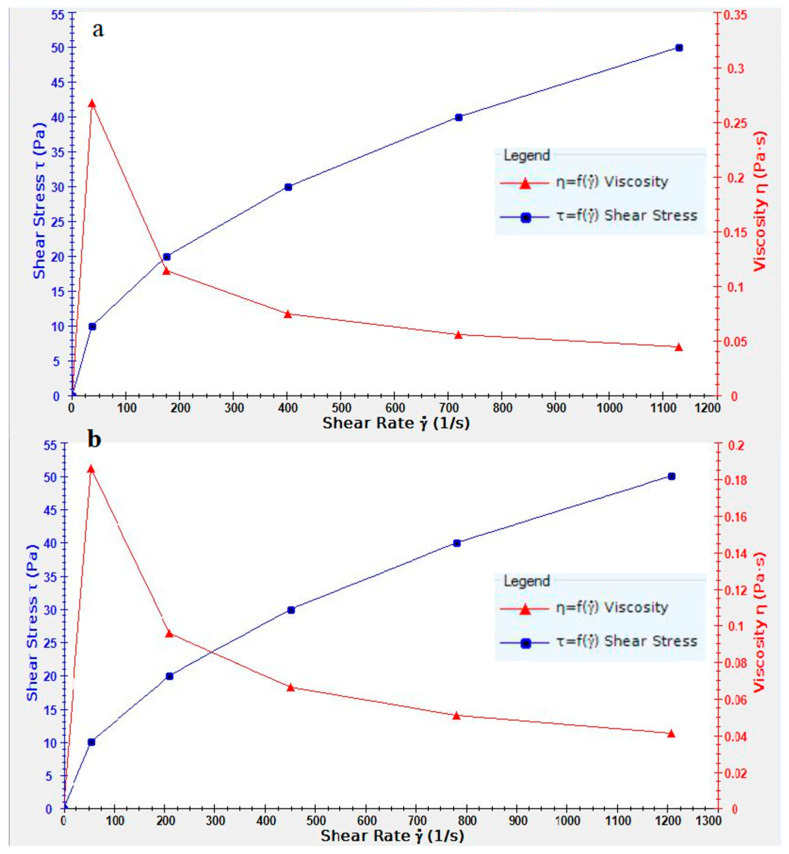
Viscosity profiles for the solutions: (**a**) CS-PEO 2.5% and (**b**) CS-PEO 2.5% loaded with Buforin I at 20× MBC. The red curve represents viscosity as a function of shear rate, while the blue curve depicts shear stress as a function of shear rate.

The results showed that the solution viscosity decreased with the increase in the shear stress in chitosan-PEO solutions containing 1, 10, and 20 times the MBC of Buforin I. This behavior shows the pseudoplastic properties of solutions (Figure 1). As presented in Table 1, the addition of Buforin I did not affect the viscosity of the electrospinning solutions. As the Buforin I concentration was elevated, the electrical conductivity of the samples lowered significantly (*p* < 0.05), but pH remained unchanged (Table 1).

### 3.2. Examination of Nanofibrous Membrane Morphology

**Figure 2:**
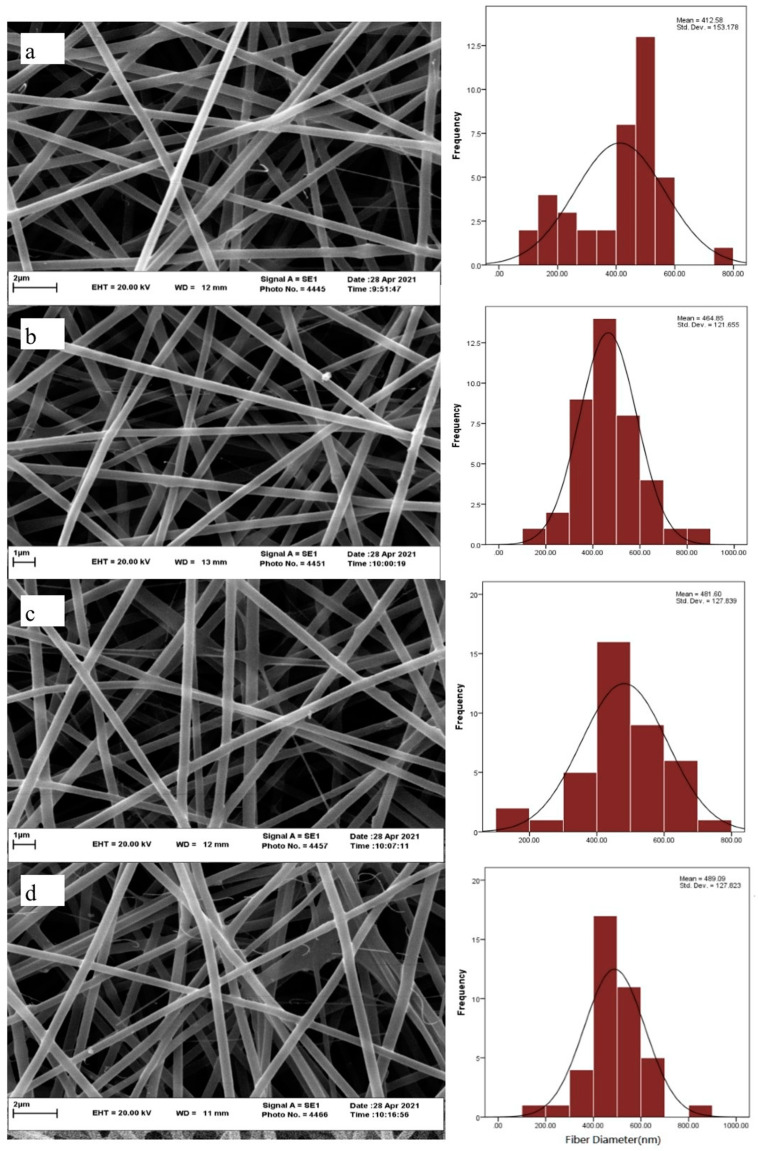
SEM images and average diameters of nanofibers for (**a**) CS-PEO 2.5%, (**b**) CS-PEO 5%, (**b**) CS-PEO 2.5% loaded with Buforin I at MBC, (**c**) CS-PEO 2.5% loaded with Buforin I at 10× MBC, and (**d**) CS-PEO 2.5% loaded with Buforin I at 20× MBC.

As depicted in Figure 2, the smallest diameter belonged to the CS-PEO nanofibers, and no significant change was observed in the nanofiber diameters after the addition of the peptide up to 10× MBC. At the same time, as the peptide concentration increased from 10 to 20 times MBC, the diameters were significantly enlarged (*p* < 0.05). This could be due to the higher electrical conductivity of the CS-PEO solution than that of the CS-PEO one loaded with the peptide, because the higher the electrical conductivity, the thinner the fibers. It is known that with a rise in a solution’s electrical conductivity, the jet load density increases; as a result, the solution carries more electrical charge and is more stretched while being injected from the syringe. Consequently, thinner fibers are produced. The literature shows that CS-PEO nanofibers typically have diameters varying from 200 to 400 nm and not exceeding 475 nm, conforming to the findings of the present study [26,27]. This implies that the production of the nanofibers was successful in the present study. Wang et al. (2023) reported that the incorporation of the antimicrobial peptide of Pac-525 to gelatin-CS nanofibers enlarged their diameters significantly from 359 to 409 nm [28].

### 3.3. Differential Scanning Calorimetry of Nanofibrous Membranes

The thermograms of Buforin I and the nanofibers of CS-PEO and CS-PEO-Buforin I are illustrated in Table 2. The DSC properly revealed that the CS-PEO nanofibers were loaded with Buforin I. In the thermogram of Buforin I, the endothermic peak at 71.12 °C is associated with the peptide melting point, and the exothermic one at 241.15 °C is related to its destruction. In the thermogram of the CS-PEO nanofibers, two endothermic peaks were observed; the first one at 54.90 °C corresponds to water evaporation and crystallization of PEO, and the second one at 178.87 °C corresponds to the glass transition temperature of nanofibers (Tg). The melting point of the CS-PEO nanofibers was elevated from 54.90 to 65.81 °C, owing to the addition of the peptide. Additionally, the peak related to the crosslinks was displaced from 135.22 to 141.57 °C, demonstrating the presence of the peptide in the CS-PEO nanofibers. The disappearance of the peptide peaks in the thermogram of the peptide-loaded nanofibers revealed the distribution of the peptide molecules within the amorphous phase of the biomaterials. Another point understood from the comparison of the thermograms was the decrease in enthalpy from −280.14 to −834.84 mJ in the temperature range of 214–215 °C as a result of the addition of the peptide to the CS-PEO nanofibers. This indicated the increase in the thermal stability of the biopolymer.

### 3.4. Fourier-Transform Infrared Spectroscopy of Nanofibrous Membranes

**Figure 3:**
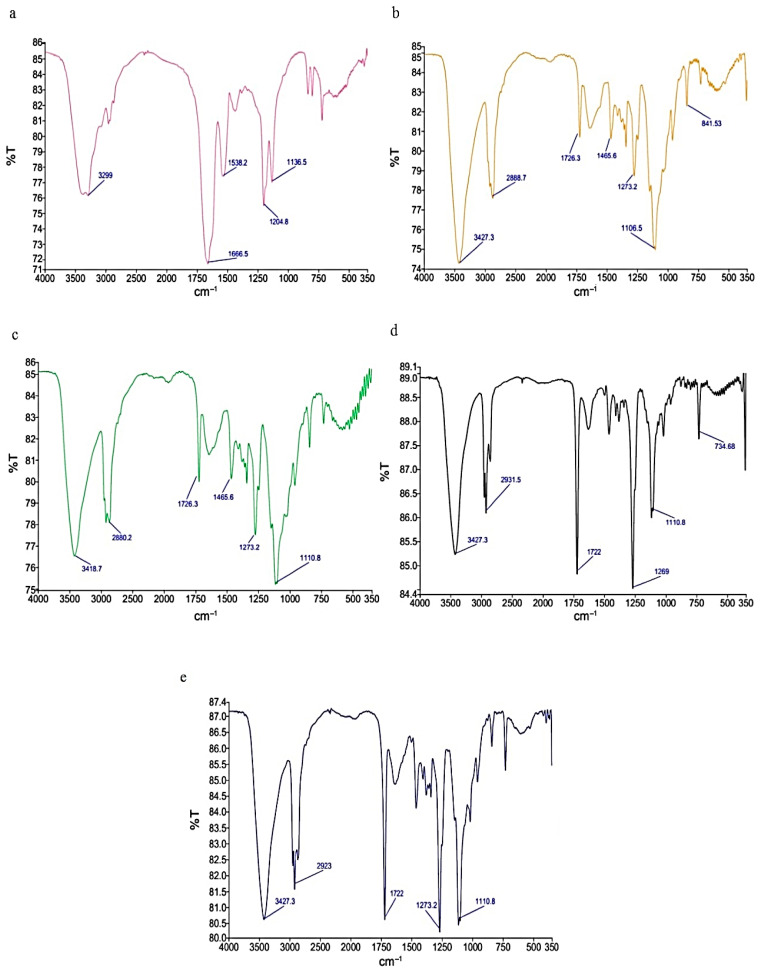
FTIR spectra of nanofibers of (**a**) Buforin I, (**b**) CS-PEO 2.5%, (**c**) CS-PEO 2.5% loaded with Buforin I at MBC, (**d**) CS-PEO 2.5% loaded with Buforin I at 10× MBC, and (**e**) CS-PEO 2.5% loaded with Buforin I at 20× MBC.

FTIR was utilized to identify the functional groups and determine the type of bonds formed between CS-PEO and Buforin I in the nanofibers. Figure 3 depicts the spectra of Buforin I and the nanofibers of CS-PEO and CS-PEO-Buforin I. The results showed the emergence of a band (C=O bending) in the wavenumber range of 600–650 cm^−1^ in the CS-PEO-Buforin I nanofibers compared with the CS-PEO ones. This band was also observed in the spectrum of the peptide. Furthermore, a peak was seen at 1000–1250 cm^−1^, associated with the stretching vibration of etheric C-O groups, in the nanofibers of CS-PEO-Buforin I and CS-PEO. However, it did not appear in the spectrum of Buforin I. The broad band in the peptide spectrum at 1650–1700 cm^−1^, resulting from the stretching vibration of C=O groups (80%), C-N stretch (10%), and N-H bending vibration (10%), was shorter and narrower in the spectra of the nanofibers. In conclusion, the FTIR findings proved the loading of Buforin I in the CS-PEO nanofibers. Similar results have been reported by Yu et al. (2022) for the antimicrobial nanohybrid based on CS and the antimicrobial peptide of microcin J25. They declared that the loading of the peptide in the CS-containing nanoparticles changed the FTIR spectra, and the variations in the band related to amine type II in the CS structure were observed in the range of 3310–3350 cm^−1^ after the loading of the peptide [29].

### 3.5. X-Ray Diffraction of Nanofibrous Membranes

**Figure 4:**
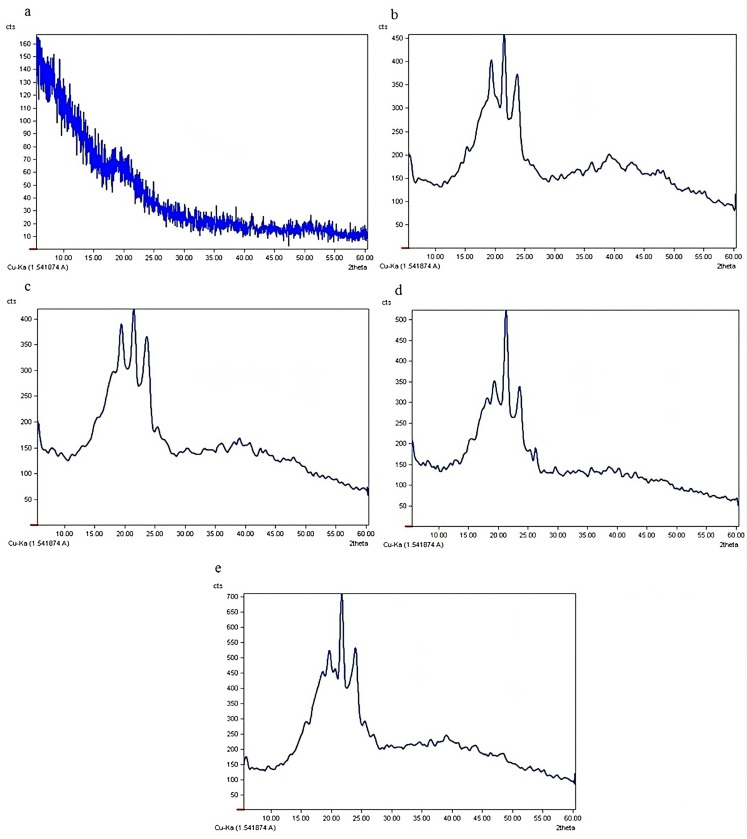
Diffraction patterns of nanofibers of (**a**) Buforin I, (**b**) CS-PEO 2.5%, (**c**) CS-PEO 2.5% loaded with Buforin I at MBC, (**d**) CS-PEO 2.5% loaded with Buforin I at 10× MBC, and (**e**) CS-PEO 2.5% loaded with Buforin I at 20× MBC.

XRD is a useful tool for the determination and measurement of crystalline structures. The crystalline structure of nanofibers depends on the properties of the solvent and polymer used [29,30]. The diffractogram of Buforin I has a relatively sharp peak at 2θ ≈ 20°, indicating the crystalline structure of its α-helix (Figure 4). In the diffraction pattern of the CS-PEO nanofibers, three peaks were observed in the 2θ range of 20–25°, probably associated with the inter- and intra-molecular hydrogen bonds of the functional groups of CS and PEO. The diffractogram of the CS-PEO nanofibers loaded with Buforin I at MBC had no difference from that of the CS-PEO ones, which is probably because of the very low loading of the peptide at this concentration.

In the diffractograms of the CS-PEO nanofibers loaded with Buforin I at 10× MBC and 20× MBC, the peak at 2θ ≈ 20° was less intense and almost disappeared. On the other hand, the peak at 2θ ≈ 23° was intensified in these diffraction patterns. Hosseini et al. (2021) [31] claimed that as the concentration of rosemary essential oil was raised from 0 to 10% in zein-cyclodextrin nanofibers, the XRD peaks were intensified. They attributed this to the change in the molecular structure of zein, in addition to the increase in the molecular aggregates and the structural change in the cyclodextrin α-helix as a result of the reaction with rosemary essential oil. Other researchers have achieved similar results in the case of protein-based nanofibers [32], polysaccharide films [33], and polysaccharide nanofibers [34].

### 3.6. Mechanical Strength of Nanofibrous Membranes

The tensile and bending strengths of the CS-PEO nanofibers before and after being loaded with the different concentrations of Buforin I were computed using the force–displacement curves and Young’s modulus (Table 3). The highest tensile strength belonged to the CS-PEO nanofibers loaded with Buforin I at 20× MBC, which was significantly (*p* < 0.05) higher than that of the CS-PEO ones. The higher the Young’s modulus, the higher the tensile strength. The tensile strengths of the CS-PEO nanofibers loaded with Buforin I at MBC and 10× MBC did not significantly differ from those of the CS-PEO ones. The FTIR results revealed that the OH and NH_2_ groups of Buforin I bound with the functional groups of CS and PEO through hydrogen bonds. Moreover, the XRD and DSC results confirmed the distribution of the peptide in the nanofibers. The formation of these bonds could be a reason behind the higher mechanical strength of the nanofibers loaded with Buforin I at 20× MBC, relative to the peptide-free CS-PEO nanofibers. It should also be noted that no range has ever been presented for mechanical properties to accept or reject fibers. Zhu et al. (2021) investigated the fibers containing ropinirole (one of the most important drugs for curing Parkinson) with the ability to dissolve in saliva. They mentioned that they could not determine a reasonable range for the fibers’ mechanical properties, which could only be employed to compare various fibers [35,36].

### 3.7. Peptide Cumulative and Kinetic of the Buforin I Release from Nanofibrous Membranes

**Figure 5:**
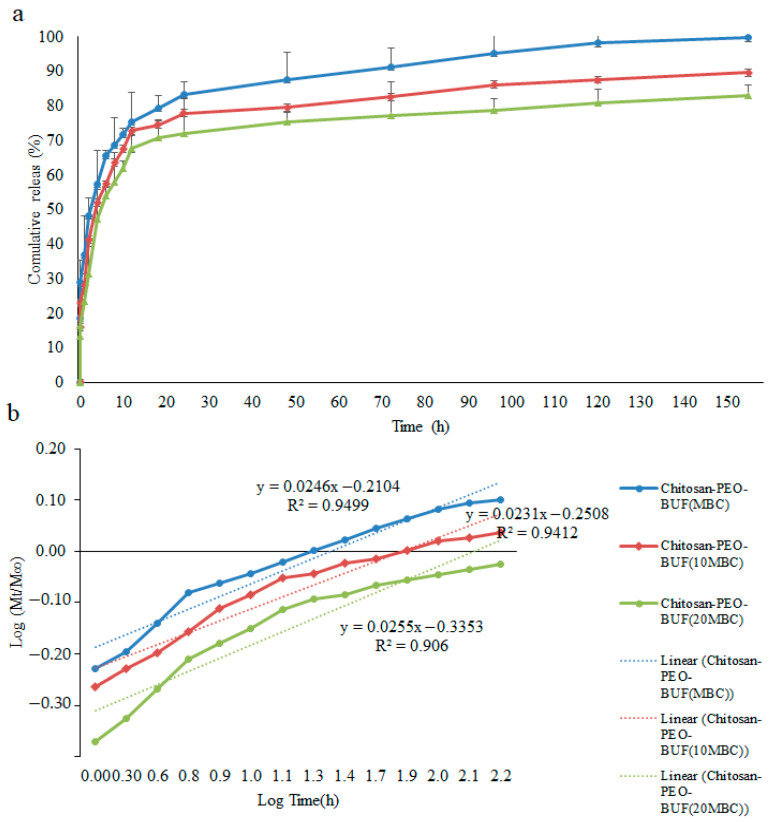
(**a**) Cumulative release of Buforin I; (**b**) cumulative release of Buforin I from CS-PEO nanofibers. PEO: poly ethylene oxide; BUF: Buforin I.

As shown in Figure 5a, Buforin I was rapidly released from the nanofibers in the early hours (20 h). Afterward, the release continued constantly at a very low rate for 6 days. The initial rapid release developed the initial effective concentration, brought the antimicrobial effect forward, and caused the effect to continue as the release endured. A comparison between the inhibition zone and the peptide release profile indicated the agreement between the results of the two tests. Consistent with these findings, Su et al. (2020) mentioned that the release of engineered human cathelicidin peptides loaded in pluronic nanofibers followed a burst trend in the first 8 days and subsequently continued slowly for 4 weeks [36]. Numerous compounds, including antibiotics, anticancer drugs, proteins, and growth factors, have been loaded in nanofibers using electrospinning, and their release behavior has been investigated. Hernández-Rangel et al. (2020) [37] cited that the release of lysozymes from polycaprolactone-PEO nanofibers exploded at first and subsequently endured at a slow rate. They reported that the highest lysozyme release from the nanofibers (87%) occurred during 12 days [37]. Hajji et al. (2022) declared that CS-gelatin nanofibers loaded with the nanoparticles of hydroxyapatite (nHAp) and the antimicrobial peptide of Pac-525 had a long-term release for 1 month and were capable of restraining microbial growth during this period [38].

The percentage of cumulative release in PBS buffer decreased (*p* > 0.05) with a rise in the Buforin I concentration in the CS-PEO nanofibers from MBC to 20× MBC. In general, the release amount of compounds from nanofibers depends on the number of fiber layers and the total amount of the loaded compound [36]. Similar results have been reported by Kurečič et al. (2019) [39]. They maintained that as the concentration of nonsteroidal anti-inflammatory drugs (NSAIDs) was elevated from 3 to 10% in carboxymethyl cellulose–polyethylene glycol nanofibers, the release percentage was significantly reduced. The decrease in the total release percentage as a function of concentration may be associated with the porous structure of the nanofibers [39]. It is suggested that an increase in specific surface area and network structure density, in addition to a decrease in pore size, restricted the diffusion and dissolution of Buforin through the cross-linked nanofibrous network of CS/PEO.

The Korsmeyer–Peppas equation is a well-known method for studying the kinetics of releasing Buforin I. If the release behavior is consistent with the Korsmeyer–Peppas release kinetics (Figure 5b), a linear relationship between log(Mt/M∞) and log(t) must be present. The release exponent (n) is determined by the slope of the line, which can be used to determine the release mechanism. The Korsmeyere–Peppas model utilized in this study fits the release data with close approximation, as indicated by the high amount of R-squared values for all three formulations. The value of the release exponent (n) obtained for MBC, 10× MBC, and 20× MBC formulations was 0.024, 0.023, and 0.025, respectively.

### 3.8. Antimicrobial Activity of Nanofibrous Membranes

So far, various studies have examined the antimicrobial activity of CS-PEO nanofibers loaded with different active substances [29,36]. The results demonstrated that the inhibition zone diameter was significantly enlarged by adding the peptide to the CS-PEO nanofibers (Table 4). In the case of the nanofibers loaded with Buforin I at 10× MBC, the inhibition zone diameter was not significantly different from that of the commercial antibiotic disk. Nevertheless, the inhibition zone diameter at 20× MBC was significantly higher than that of the commercial antibiotic disk. Additionally, the mean comparison showed that the nanofibers loaded with Buforin I at 20× MBC had the longest inhibition zone diameter for all the microorganisms tested, which was significantly (*p* < 0.05) longer than that of the commercial antibiotic disk. The nanofibers loaded with human-engineered cathelicidin were able to kill *K. pneumonia* and *Acinetobacter baumannii* with high efficiency [36]. Yu et al. (2022) [29] declared that the nanohybrid of CS and the antimicrobial peptide of microcin J25 produced a remarkable antimicrobial effect on both Gram-negative and Gram-positive bacteria. Moreover, the microcin nanoparticles showed their inhibitory effect on Gram-negative and Gram-positive bacteria at lower concentrations, compared with free microcin. The electrospun nanofibers of silk fibroin loaded with the antimicrobial peptide of cecropin B could reduce the number of *E. coli* (93.5%) and *S. aureus* (91.6%) inoculated onto their surfaces after 2 h [40]. The PEO nanofibers loaded with plantaricin 423 bacteriocin prevented the growth of *Enterococcus faecium* and *Lactobacillus sakei*. Hajji et al. (2022) [38] claimed that the CS-gelatin nanofibers loaded with nHAp nanoparticles and the antimicrobial peptide of Pac-525 could restrain the growth of *E. coli* and *S. aureus*.

### 3.9. Cell Toxicity and Hemolytic Activities of Nanofibrous Membranes

**Figure 6:**
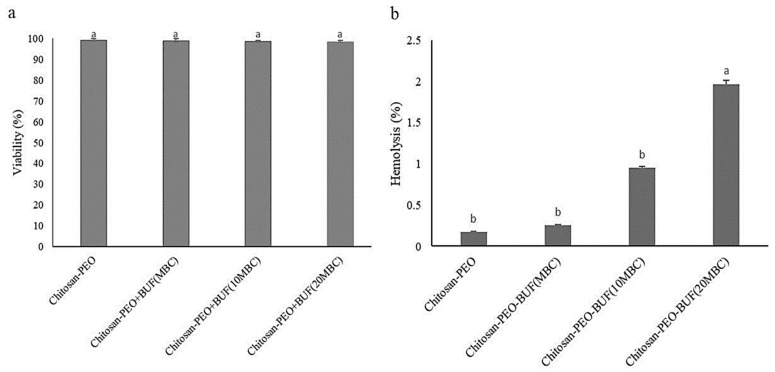
(**a**) Cell toxicity and (**b**) hemolytic activities of nanofibers of CS-PEO 2.5%, CS-PEO 2.5% loaded with Buforin I at MBC, CS-PEO 2.5% loaded with Buforin I at 10× MBC, and CS-PEO 2.5% loaded with Buforin I at 20× MBC on human fibroblast cell line. Different letters demonstrate significant differences (*p* < 0.05).

The results revealed that the nanofibers had no impact on the viability of the human fibroblast cell line (Figure 6a) and did not show any hemolytic activity (Figure 6b).

The cationic nature of antimicrobial peptides enables them to identify the target cell [36] reported that the nanofibers loaded with human-engineered cathelicidin, which had been used for the treatment of biofilms on diabetic chronic wounds, had no toxic effect on skin cells and monocytes. Furthermore, wound coverage in the mice induced with type II diabetes appropriately prevented biofilm formation.

The cationic nature of AMPs principally contributes to cell selectivity, as the surface of bacterial membranes is more negatively charged relative to mammalian cells [1]. The membrane of a bacterial cell is rich in acidic phospholipids, including phosphatidylglycerol and cardiolipin [1]. The bacterial cells with larger amounts of negatively charged lipids are more sensitive to AMPs [4]. The cell walls also consist of anionic molecules like lipopolysaccharides in the outer membrane of Gram-negative bacteria and teichoic acids and lipoteichoic acids in the peptidoglycan of Gram-positive ones. On the other hand, acidic phospholipids are normally sequestered in the inner leaflets of plasma membranes of mammalian cells [5]. The outer leaflets majorly comprise zwitterionic phosphatidylcholine and sphingomyelin, even though negatively charged gangliosides exist as minor species. Lee et al. (2008) [41] indicated that gangliosides have a key role in the cellular entry of the Buforin IIb peptide. In addition to the cell surface charge, some other factors also contribute to cell selectivity. The membrane-stabilizing cholesterol in mammalian cells protects the cells from attack by AMPs. An inside-negative transmembrane potential facilitates membrane permeabilization, probably by facilitating the insertion of positively charged peptides into the membranes. The transmembrane potential of bacterial cells is more negative than that of normal mammalian cells [1].

### 3.10. Evaluation of the Effects of Electrospun Buforin I-Loaded Nanofibrous Membrane on Beef Shelf Life at Refrigerator Temperature

#### 3.10.1. pH, a_w_, and Weight Loss Values

pH is a crucial factor in meat products, as it affects the occurrence and distribution of microorganisms. It shows values below 6 in fresh meats. The pH value of the uncoated beef and beer coated with CS-PEO nanofibers without the Buforin I sample showed a significant increase (*p* < 0.05) from 5.89 to 6.35 and from 5.86 to 6.31, respectively, during storage (Table 4). The initial pH values were already higher than 5. The pH values increased significantly during storage (*p* < 0.05). Beef coated with CS-PEO nanofibers and Buforin I showed maximum values on day 9. At the end of storage, the coated samples obtained significantly lower values than the uncoated beef (*p* < 0.05). The production of trimethylamines by the activity of microorganisms and the accumulation of alkaline substances such as biogenic amines and ammonia may be the main reason for the increase in pH during storage [42]. These results are in agreement with those found by Aguirre-Guataqui et al. (2022) [43], Carvajal et al. (2022) [44], and Muñoz et al. (2022) [45], indicating that Buforin could significantly (*p* < 0.05) decrease pH fluctuations [43,44,45]. Also, Alirezalu et al. (2021) showed that an ε-polylysine coating with a stinging nettle extract in edible films reduces pH during storage [46].

Water activity (a_w_) is one of the important parameters for chemical, microbial, and enzymatic activities. This research showed that the coating containing Buforin I improves the shelf life of fresh meat cuts by reducing a_w_ and lipid oxidation. The reduction in drip loss in beef and binding water that would otherwise be available for microbial growth causes slowed microbial growth (Table 5). Economou et al. (2022) [47] constructed edible chitosan effective against Listeria monocytogenes and *Staphylococcus aureus* in beef cuts. The results showed that this edible chitosan membrane reduced microbial growth by decreasing a_w_.

CS-PEO nanofibers with Buforin I had a higher surface tension compared to CS-PEO nanofibers without Buforin I. They could act as a barrier against water due to their surface activity. Water diffusion in beef coated with CS-PEO nanofibers containing Buforin I was significantly reduced (Table 5). Moreover, the lower water vapor permeability of the coating solutions compounds reduced water loss through evaporation from the beef. Therefore, the weight loss of the treated samples was reduced compared to the control sample during the storage process. Zou et al. (2022) [48] and Yan et al. (2024) [49] showed that gelatin–chitosan edible films acted as moisture-sacrificial agents instead of moisture barriers by losing their water content. Therefore, the weight loss of the coated beef was delayed.

Fresh beef loses weight due to water evaporation, which leads to the loss of nutrients such as sarcoplasmic proteins and water-soluble vitamins in the beef. The weight loss rate of the samples increased during storage. The uncoated sample experienced the highest weight loss of 16.43% after 9 days, which was twice as much as the beef coated with CS-PEO nanofibers with Buforin I, and showed a significant difference (*p* < 0.05). The amount of weight loss in the sample coated with CS-PEO nanofibers with Buforin I was lower than the control sample and the sample coated with CS-PEO nanofibers without Buforin I. The weight loss was less than 8 and 10% in 9 days for the coated beef with Buforin I-loaded nanofibers and the sample coated with CS-PEO nanofibers without Buforin I compared to the uncoated beef. This indicates that the Buforin I-loaded nanofibers had the barrier ability to effectively prevent the capillary phenomenon of water flow and keep more moisture on the sample surface, thus improving the freshness and texture of the beef. This edible film can be a good barrier for oxygen and carbon dioxide, which leads to a decrease in enzyme activity. Our result is consistent with the findings of Yan et al. (2024) [49]; gelatin–chitosan–Cyclocarya paliurus flavonoid-based films slowed the weight loss of chilled beef compared to the control. Similar results were reported by Wang et al. (2017) [22] and Gedarawatte et al. (2021) [50].

#### 3.10.2. Texture Profile Analysis and Measurement of the Thiobarbituric Acid

**Figure 7:**
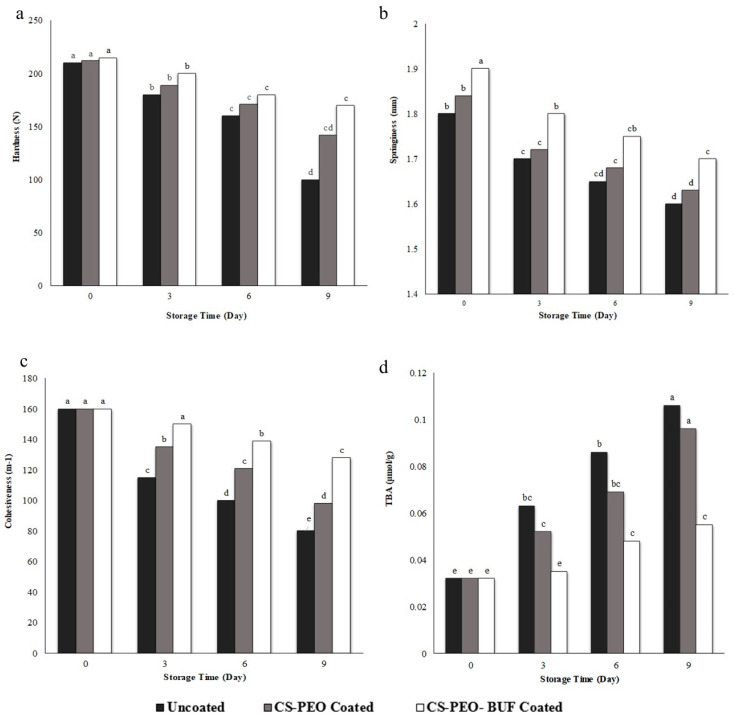
Evolution of the effects of electrospun Buforin I-loaded nanofibrous membranes on the texture profile included: (**a**) Hardness, (**b**) Springiness, (**c**) Cohesiveness, and (**d**) Thiobarbituric acid amount of beef samples during storage (4 °C). CS-PEO-BUF: coated with Buforin I-loaded CS-PEO nanofibers; CS-PEO: coated with CS-PEO nanofibers without Buforin I. Different lowercase letters indicate a significant difference (*p* < 0.05).

Beef texture is an important indicator of its freshness. The degree and distribution of fat affect the hardness of meat. Tenderness in meat can be maintained by intramuscular fat by dividing and diluting the connective tissues. The fat content decreases, resulting in a decrease in hardness [51]. The hardness value decreased due to lipid oxidation in the beef samples, which could cause muscle tissue to soften during storage [52]. Beef cohesiveness refers to the strength of the internal bonds that build its structure. It directly relates to beef’s tensile and compressive strength [53]. The greatest decrease in beef hardness, springiness, and cohesiveness was observed in the uncoated samples. The uncoated sample and beef coated with CS-PEO nanofibers without Buforin I showed a significant (*p* < 0.05) reduction in cohesiveness from 160 to 80 m^−1^ and 160 to 98 m^−1^, respectively, over the storage period. Beef coated with CS-PEO nanofibers with Buforin I showed a cohesiveness of 128 m^−1^ on the ninth day of storage (Figure 7). Adding Buforin I-loaded nanofibers to the film prevented proteins from oxidizing in the beef samples, thus preserving the fresh beef’s hardness. Beef coated with CS-PEO nanofibers with Buforin I had better water retention than the beef coated with CS-PEO nanofibers without Buforin I and the uncoated samples. In general, coated beef exhibited better texture properties than the control sample. All meat samples experienced a decrease in hardness as storage time increased, as shown in Figure 7a. Springiness is achieved by a network structure formed by the protein hydration layer in meat that can resist external forces. It is defined by the degree of deformation and recovery after the applied force is removed. The springiness of all samples changed significantly during 9 days of storage (Figure 7b). The results showed that CS-PEO-Buforin I coatings increased shelf life and improved the texture properties of beef during storage. This coating delays lipid oxidation and preserves meat quality. Guerrero et al. (2015) [54] reported that a soy protein coating effectively delayed lipid oxidation and the deterioration of beef quality during storage. The textural parameters of the samples coated with soy protein were maintained for up to 14 days, in contrast to the deterioration observed in the control group. Cheng et al. (2021) [55] showed that chitosan-based coatings combined with ε-polylysine and glutathione enhanced the shelf life and improved texture characteristics of beef slices during storage. Yan et al. (2024) [49] found that the control samples had the highest hardness and reduced the chewiness of beef, while the gelatin–chitosan–0.3% Cyclocarya paliurus flavonoid film samples showed higher hardness and chewiness than other treatments during storage. Our findings correspond to those obtained by Zhang et al. (2021) [11] and Gedarawatte et al. (2021) [56].

The unsaturated fatty acids in the fat can decompose when exposed to oxygen, resulting in malondialdehyde, a cytotoxic product. Generally, the TBARS value of fresh beef is between 0.20 and 0.66 μmol/g. All samples had an initial TBARS value of 0.32 μmol/g. The TBA values of treatments increased during storage (Figure 7d). The TBA values of the control samples were significantly higher than those of the coating treatments after 3 days. The Buforin I-loaded nanofibers had a significant impact on the increase in TBA. On the 9th day, the TBA value of the uncoated sample, sample coated with CS-PEO nanofibers without Buforin I, and sample coated with CS-PEO nanofibers with Buforin I, were 0.106 μmol/g, 0.096 μmol/g, and 0.055 μmol/g, respectively. The results showed that the Buforin I-loaded nanofibers delay the fat in the beef samples from oxidizing, resulting in a better preservation effect. This is due to the antibacterial abilities of Buforin I. Bermúdez-Oria et al. (2019) [57] showed that the pectin–fish gelatin film containing hydroxytyrosol-dihydroxyphenylglycol antioxidants reduced the formation of oxidation products in the form of TBA in beef coated compared to a film without antioxidants. Cardoso et al. (2019) [58] reported that a chitosan–gelatin coating effectively slowed lipid oxidation and microbial growth in beef steaks and extended the shelf life from 6 to 10 days. Moreover, the addition of tomato antioxidants to gelatin films has been shown to effectively prevent the increase in TBA values of pork meat during storage [58], as demonstrated by similar studies [59]. These findings correspond to those obtained by Alirezalu et al. (2021) [46] who evaluated the effect of an ε-polylysine coating with a stinging nettle extract for fresh beef preservation.

#### 3.10.3. Color Analysis

Consumers use the color of beef to assess freshness, which can influence purchasing behavior. The color of the meat can be affected by the optical properties of the edible coating, which are dependent on the materials used to make it [55]. The highest and lowest increase in L* value was observed in the uncoated sample and the sample coated with CS-PEO nanofibers with Buforin I, respectively. The increase in L* value could be attributed to the beef’s increased oxidation during storage. Breaking the peptide sequence leads to an aggregation phenomenon that modifies the amino acid chain. The brightness of beef is enhanced due to changes in protein conformation and structure. The loss of muscle protein function and change in meat properties can be attributed to the formation of protein cross-links and protein carbonylation [60].

Better color and higher quality can be achieved by having a higher red index. The production of oxymyoglobin and oxyhemoglobin, as well as the formation of metmyoglobin and methemoglobin, caused the a* values of all samples to decrease after day 3 due to the brown color produced by these proteins. According to the results, the red color of the coated beef decreased more slowly than that of the control samples. The uncoated sample and beef coated with CS-PEO nanofibers without Buforin I showed a significant (*p* < 0.05) reduction in a* value from 5.5 to 2.44 and 5.5 to 2.93 during the storage period, respectively. The a* value of beef coated with CS-PEO nanofibers with Buforin I was 4.03 on the ninth day of storage (Table 6). It is possible that the edible coating prevented the contact of beef with oxygen.

Also, the results showed that the b* value of beef coated with CS-PEO nanofibers with Buforin I did not change significantly during storage; however, it increased in the uncoated sample and sample coated with CS-PEO nanofibers without Buforin I over time (Table 6). The highest b* value (16.7) was observed in the uncoated sample in 9 days, because of the formation of hydrogen sulfide by enzymes and microorganisms that degrade proteins. When hydrogen sulfide combines with hemoglobin, it forms a yellow complex, thus increasing the value of b*. Since the edible coating prevented oxygen from reaching the beef, the b* values of the coated samples were lower than the uncoated beef. As a result, nanofibers can preserve and intensify the beef’s red color while extending its shelf life. Guerrero et al. (2015) [54] showed that protein-based edible coatings improved the surface color stability of meat during storage. Cheng et al. (2021) [55] mentioned that chitosan-based coatings combined with ϵ-polylysine and glutathione maintained a red index in beef slices. Alirezalu et al. (2021) [46], Gedarawatte et al. (2021) [50], and Zhang et al. (2021) [11] reported little change in the b* value of coated beef.

#### 3.10.4. Microbiological Analysis

Beef has a high nutritional value and is sensitive to microbial contamination. The total count of aerobic bacteria in all samples increased during storage. The total count of beef coated with CS-PEO nanofibers with Buforin I was less than the other sample. The total count of the beef coated with CS-PEO nanofibers with Buforin I during storage was below 3 log CFU/g, but the uncoated sample exhibited the most noticeable increase in the overall number of aerobic bacteria. The initial total count of the uncoated sample was 1.2 log CFU/g; then, after 9 days, it reached 7.2 log CFU/g (Table 7). Since Buforin I-loaded nanofibers have a bacteriostatic effect, they prevent the proliferation of microorganisms in beef samples. Naseri et al. (2020) [61] found that a gelatin–chitosan–Hornbeam Ferula film prevented microbial growth and extended the shelf life of meat. Yan et al. (2024) [49] reported that the initial total viable count of the uncoated beef was 2.3 log CFU/g and reached 6.28 log CFU/g after 14 days of storage, and the total viable count of the beef coated with Cyclocarya paliurus flavonoid was below 6 log CFU/g during storage. Cheng et al. (2021) [55] reported similar results while investigating the effect of chitosan-based coatings on the microbial characteristics and the shelf life of beef slices during refrigerated storage. Coating the beef with Buforin I-loaded nanofibers decreased the bacterium, which was similar to pathogenic staphylococci and enteric bacteria, during storage. Over time, bacteria levels in the beef coated with CS-PEO nanofibers with Buforin I were lower than in the uncoated sample and beef coated with CS-PEO nanofibers without Buforin I (Table 7). This was due to the increased antibacterial activity of the coatings during storage. Azarifar et al. (2020) [62] found that chitosan-coated meat had significantly lower counts of *Staphylococcus*, *Enterobacteriaceae*, and *E. coli* compared to uncoated and gelatin-coated meat. Similar results were reported by Esmaeili et al. (2021) [63], who investigated the effect of a chitosan–Lepidium satium seed gum composite coating and nano-coating on the microbial quality of beef.

## 4. Conclusions

To extend the resistance of Buforin I to environmental conditions and control its release, electrospinning was conducted using CS and PEO. The peptide was encapsulated at MBC, 10 MBC, and 20 MBC. The FTIR results showed that the OH and NH_2_ groups of Buforin I bound with the functional groups of CS and PEO through hydrogen bonds. Furthermore, the XRD and DSC findings confirmed the peptide distribution within the nanofibers. The formation of such bonds led to the elevation of the mechanical strength of the Buforin I-loaded nanofibers compared with the peptide-free CS-PEO nanofibers. The release of Buforin I from the nanofibers occurred extremely rapidly in the early hours. Afterward, it constantly endured at a very low rate for 6 days. The inhibition zone diameter was significantly elongated after the incorporation of the peptide into the nanofibers. The results of the cell toxicity examination demonstrated that the nanofibers did not affect the viability of the human fibroblast cell line. In conclusion, these nanofibers can be used as a part of antimicrobial packages or as antimicrobial membranes to prevent or delay food spoilage. In addition, these nanofibers can be applied in covering burn wounds and chronic wounds caused by some diseases like type II diabetes. Also, the results showed that the Buforin I-loaded nanofibers had a good antioxidant and antimicrobial effect on beef and its formulation does not affect the organoleptic properties of the product.

## Figures and Tables

**Table 1:** The viscosity, electrical conductivity, and pH of CS-PEO solution before and after being loaded with Buforin I at MBC, 10× MBC, and 20× MBC.

Sample	Viscosity (Pa·s)	Electrical Conductivity (µS/cm)	pH
CS/PEO	0.0443 ± 0.03 ^a^	663 ± 19.3 ^a^	1.81 ± 0.2 ^a^
CS/PEO/Buforin I (MBC)	0.0425 ± 0.13 ^a^	627 ± 17.5 ^a^	1.79 ± 0.13 ^a^
CS/PEO/Buforin I (10× MBC)	0.0429 ± 0.14 ^a^	605 ± 20.2 ^b^	1.76 ± 0.15 ^a^
CS/PEO/Buforin I (20× MBC)	0.0414 ± 0.016 ^a^	596 ± 15.3 ^c^	1.76 ± 0.27 ^a^

MBC: minimum bactericidal concentration; CS: chitosan; PEO: poly ethylene oxide. Different lowercase letters indicate a significant difference (*p* < 0.05).

**Table 2:** Thermogram data of Buforin I, CS-PEO 2.5%, and (c) CS-PEO loaded with Buforin I.

Samples	T1_(peak1)_ (°C)	T2_(peak2)_ (°C)	T2_(peak3)_ (°C)	Enthalpy (j/g)
Buforin I	71.12	241.15	-	−574.69
CS-PEO	54.90	178.87	-	−280.14
CS-PEO-BUF I	65.81	139.32	215.38	−834.84

CS: chitosan; PEO: poly ethylene oxide, BUF I: Buforin I.

**Table 3:** Tensile test parameters of nanofibers of CS-PEO 2.5% and CS-PEO 2.5% loaded with Buforin I at MBC, 10× MBC, and 20× MBC.

Nanofiber	Diameter (mm)	Yield Stress (MPa)	Maximum Force (N)	Young’s Modulus (MPa)
CS/PEO	0.22 ± 0.049 ^a^	1.923 ± 0.2 ^b^	4.465 ± 0.180 ^ab^	81.95 ± 7.27 ^b^
CS/PEO/Buforin I (MBC)	0.20 ± 0.012 ^a^	1.332 ± 0.13 ^b^	3.085 ± 0.403 ^b^	68.90 ± 10.75 ^b^
CS/PEO/Buforin I (10× MBC)	0.21 ± 0.012 ^a^	1.515 ± 0.15 ^b^	2.850 ± 0.028 ^b^	77.55 ± 1.63 ^b^
CS/PEO/Buforin I (20× MBC)	0.21 ± 0.49 ^a^	2.953 ± 0.27 ^a^	6.535 ± 0.516 ^a^	101.55 ± 14.06 ^a^

MBC: minimum bactericidal concentration; CS: chitosan; PEO: poly ethylene oxide. Different lowercase letters indicate significant difference (*p* < 0.05).

**Table 4:** Inhibition zone diameters of 8 mm antibiotic disks and nanofibers of CS-PEO 2.5%, CS-PEO 2.5% loaded with Buforin I at MBC, CS-PEO 2.5% loaded with Buforin I at 10× MBC, and CS-PEO 2.5% loaded with Buforin I at 20× MBC.

Microorganisms	IZD (mm)				
	Antibiotic Disk	Chitosan-PEO	Chitosan-PEO + BUF (MBC)	Chitosan-PEO + BUF (10× MBC)	Chitosan-PEO + BUF (20× MBC)
*S. aureus*	19.50 ± 0.70 b	8.5 ± 0.70 c	14.00 ± 1.47 c	18.00 ± 0.20 bc	24.00 ± 1.41 a
*L. monocytogenes*	21.00 ± 10.41 ab	8.0 ± 0.00 c	15.00 ± 1.47 c	17.50 ± 0.70 bc	23.50 ± 1.51 a
*B. cereus*	24.00 ± 1.41 d	8.0 ± 0.00 c	13.00 ± 1.41 c	22.50 ± 2.12 b	29.00 ± 1.70 a
*E. coli*	21.50 ± 0.70 c	8.0 ± 0.00 d	13.65 ± 2.33 b	16.60 ± 0.84 ab	19.35 ± 0.90 a
*S. Typhi*	21.25 ± 0.35 b	8.5 ± 0.70 c	13.75 ± 1.06 c	22.80 ± 0.29 b	28.30 ± 0.99 a
*P. aeruginosa*	15.75 ± 1.06 c	8.0 ± 0.00 d	10.50 ± 0.70 d	19.25 ± 1.06 b	27.80 ± 0.30 a
*C. albicanc*	17.35 ± 0.50 b	8.3 ± 0.70 c	12.20 ± 0.29 c	16.10 ± 0.14 b	20.20 ± 1.13 a
*A. niger*	16.20 ± 0.30 bc	8.0 ± 0.00 c	14.55 ± 0.78 c	18.20 ± 0.30 ab	16.20 ± 0.30 a

IZD: inhibition zone diameter; MBC: minimum bactericidal concentration; the antibiotics used for the Gram-positive and Gram-negative bacteria and fungi were vancomycin (30 µg), tetracycline (30 µg), and nistatin (50 µg), respectively. Different letters represent significant differences (*p* < 0.05).

**Table 5:** Evolution of the effects of electrospun Buforin I-loaded nanofibrous membranes on pH, a_w_, and weight loss values of beef during storage (4 °C).

Properties	Beef	Storage Time (Day)			
		0	3	6	9
pH	UNC	5.89 ± 0.01 ^ed^	5.94 ± 0.02 ^d^	6.26 ± 0.01 ^b^	6.35 ± 0.01 ^a^
	CS-PEO	5.86 ± 0.01 ^e^	5.94 ± 0.02 ^d^	6.20 ± 0.01 ^b^	6.29 ± 0.02 ^ab^
	CS-PEO-BUF	5.85 ± 0.01 ^e^	5.90 ± 0.01 ^ed^	6.09 ± 0.02 ^c^	6.11 ± 0.01 ^c^
a_w_	UNC	0.99 ± 0.001 ^a^	0.985 ± 0.002 ^a^	0.979 ± 0.001 ^b^	0.973 ± 0.002 ^bc^
	CS-PEO	0.99 ± 0.001 ^a^	0.98 ± 0.001 ^b^	0.970 ± 0.001 ^bc^	0.965 ± 0.001 ^c^
	CS-PEO-BUF	0.989 ± 0.001 ^a^	0.977 ± 0.002 ^b^	0.967 ± 0.001 ^c^	0.94 ± 0.002 ^d^
weight loss (%)	UNC	1.8 ± 0.02 ^f^	6.67 ± 0.01 ^cd^	11.59 ± 0.02 ^b^	16.43 ± 0.01 ^a^
	CS-PEO	0.9 ± 0.02 ^g^	4.25 ± 0.01 ^de^	7.04 ± 0.02 ^c^	9.11 ± 0.01 ^bc^
	CS-PEO- BUF	0.8 ± 0.02 ^g^	3.13 ± 0.01 ^e^	5.46 ± 0.02 ^d^	7.8 ± 0.01 ^c^

CS-PEO-BUF: coated with Buforin I-loaded CS-PEO nanofibers; CS-PEO: coated with CS-PEO nanofibers without Buforin I; UNC, uncoated. Different lowercase letters indicate a significant difference (*p* < 0.05).

**Table 6:** Evolution of the effects of electrospun Buforin I-loaded nanofibrous membrane on the L*, a*, and b*values of beef samples during storage (4 °C).

Color Parameters	Beef	Storage Time (Day)			
		0	3	6	9
L*	UNC	52.83 ± 1.99 ^d^	55.25 ± 0.84 ^b^	55.79 ± 0.8 ^b^	56.98 ± 0.79 ^a^
	CS-PEO	52.02 ± 0.86 ^e^	53.18 ± 0.78 ^d^	54.92 ± 0.92 ^c^	56.73 ± 0.84 ^a^
	CS-PEO-BUF	51.21 ± 0.86 ^f^	51.67 ± 0.67 ^f^	52.84 ± 0.71 ^e^	53.95 ± 0.74 ^d^
a*	UNC	5.5 ± 0.68 ^a^	4.02 ± 0.35 ^bc^	2.71 ± 0.28 ^c^	2.44 ± 0.21 ^d^
	CS-PEO	5.5 ± 0.48 ^a^	4.69 ± 0.42 ^b^	3.57 ± 0.52 ^c^	2.93 ± 0.28 ^d^
	CS-PEO-BUF	5.4 ± 0.58 ^a^	5.31 ± 0.49 ^a^	4.25 ± 0.32 ^b^	4.03 ± 0.41 ^c^
b*	UNC	15.12 ± 0.11 ^b^	16.06 ± 0.20 ^a^	16.36 ± 0.23 ^a^	16.7 ± 0.28 ^a^
	CS-PEO	15.03 ± 0.13 ^b^	15.89 ± 0.22 ^a^	16.03 ± 0.20 ^a^	16.43 ± 0.23 ^a^
	CS-PEO-BUF	14.44 ± 0.18 ^c^	14.57 ± 0.19 ^c^	14.72 ± 0.16 ^c^	14.94 ± 0.20 ^c^

L* value indicates lightness, and a* and b* are chromaticity coordinates, CS-PEO-BUF: coated with Buforin I-loaded CS-PEO nanofibers; CS-PEO: coated with CS-PEO nanofibers without Buforin I; UNC, uncoated. Different lowercase letters indicate a significant difference (*p* < 0.05).

**Table 7:** Evolution of the effects of electrospun Buforin I-loaded nanofibrous membrane on of beef samples and microbial analysis during storage (4 °C).

Storage (Day)	Microbial Count (Log CFU/g)											
	Eathogenic Enteric			*E. coli*			Total Mesophil			Staphylococci		
	UNC	CS-PEO	CS-PEO-Buf	UNC	CS-PEO	CS-PEO-Buf	UNC	CS-PEO	CS-PEO-Buf	UNC	CS-PEO	CS-PEO-Buf
**0**	0.8 ± 0.07 ^f^	0.69 ± 0.07 ^f^	0.27 ± 0.01 ^g^	0.6 ± 0.02 ^f^	0.5 ± 0.02 ^f^	0.2 ± 0.01 ^g^	1.2 ± 0.08 ^e^	1.2 ± 0.06 ^e^	1.2 ± 0.04 ^e^	0.17 ± 0.07 ^g^	0.13 ± 0.05 ^g^	0.1 ± 0.05 ^g^
**3**	2.6 ± 0.13 ^d^	2.4 ± 0.13 ^d^	0.87 ± 0.03 ^f^	1.95 ± 0.09 ^de^	1.8 ± 0.09 ^de^	0.1 ± 0.01 ^g^	3.9 ± 0.12 ^c^	3.7 ± 0.12 ^c^	1.3 ± 0.01 ^e^	1.3 ± 0.04 ^e^	1.2 ± 0.03 ^e^	0.03 ^h^
**6**	3.5 ± 0.16 ^c^	3.28 ± 0.16 ^c^	0 ^h^	2.7 ± 0.14 ^d^	2.3 ± 0.14 ^d^	0 ^h^	5.3 ± 0.17 ^b^	5.12 ± 0.1 ^b^	1.5 ± 0.09 ^e^	1.7 ± 0.02 ^e^	1.4 ± 0.04 ^e^	0 ^h^
**9**	5.2 ± 0.2 ^b^	5.03 ± 0.2 ^b^	0 ^h^	3.9 ± 0.18 ^c^	3.6 ± 0.18 ^c^	0 ^h^	7.2 ± 0.2 ^a^	6.9 ± 0.2 ^a^	2.1 ± 0.07 ^de^	2.6 ± 0.14 ^d^	2.3 ± 0.1 ^d^	0 ^h^

CFU: colony-forming unit per gram, CS-PEO-BUF: coated with Buforin I-loaded CS-PEO nanofibers; CS-PEO: coated with CS-PEO nanofibers without Buforin I; UNC, uncoated. Different lowercase letters indicate a significant difference (*p* < 0.05).

## Data Availability

All data generated or analyzed during this study are included in this published article.
